# A novel series of metazoan L/D peptide isomerases

**DOI:** 10.1016/j.jbc.2024.107458

**Published:** 2024-06-08

**Authors:** Harvey M. Andersen, Hua-Chia Tai, Stanislav S. Rubakhin, Peter M. Yau, Jonathan V. Sweedler

**Affiliations:** 1 Beckman Institute, University of Illinois, Urbana-Champaign, Urbana, Illinois, USA; 2 Department of Molecular and Integrative Physiology, University of Illinois, Urbana-Champaign, Urbana, Illinois, USA; 3 Institute for Genomic Biology, University of Illinois, Urbana-Champaign, Urbana, Illinois, USA; 4 Department of Chemistry, University of Illinois, Urbana-Champaign, Urbana, Illinois, USA; 5 Department of Cell and Developmental Biology, University of Illinois, Urbana-Champaign, Urbana, Illinois, USA

**Keywords:** enzyme, neuropeptide, analytical chemistry, mass spectrometry (MS), peptide hormone, peptide isomerase, d-amino acid containing peptide

## Abstract

The function of endogenous cell-cell signaling peptides relies on their interactions with cognate receptors, which in turn are influenced by the peptides' structures, necessitating a comprehensive understanding of the suite of post-translational modifications of the peptide. Herein, we report the initial characterization of putative peptide isomerase enzymes extracted from *R. norvegicus*, *A. californica*, and *B. taurus* tissues. These enzymes are both tissue and substrate-specific across all three organisms. Notably, the lungs of the mammalian species, and the central nervous system of the mollusk displayed the highest isomerase activity among the examined tissues. *In vitro* enzymatic conversion was observed for several endogenous peptides, such as the tetrapeptide GFFD in *A. californica*, and mammalian neuropeptide FF in *R. norvegicus* and *B. taurus*. To understand their mode of action, we explored the effects of several inhibitors on these enzymes, which suggest common active site residues. While further characterization of these enzymes is required, the investigations emphasize a widespread and overlooked enzyme activity related to the creation of bioactive peptides.

The modification of ribosomally generated peptides into a D-amino acid-containing-peptide (DAACP) has broad implications, as many essential biological functions are dependent on modifications to primary products of translation. The incorporation of one or more D-residues into a larger polypeptide can deliver enhanced stability, proteolytic resistance, and altered binding affinities (1, 2, 3, 4, 5). Consequentially, DAACPs have been shown to play important roles in cell signaling and toxin production (6, 7, 8, 9, 10, 11, 12, 13).

In the metazoan, the production of a DAACP has been shown to occur as a post-translational modification (PTM) where the D-amino acid is generated from the corresponding L-isomer in the precursor peptide by the action of a peptide isomerase, sometimes referred to as a peptidyl-aminoacyl-L/D-isomerase (14). The only known metazoan examples are the peptide isomerases characterized in the venom of *Agelenopsis aperta* (15, 16, 17, 18), in skin secretions from the *Bombina* genus (19, 20, 21), and in the venom of monotreme mammals (11, 22, 23, 24, 25). Between these cases, a key similarity lies within the functional environment of these isomerases. Compared to isomerase activity obtained from tissues and organs, the individual protein components of venom and skin secretions offer a natural enrichment of both DAACPs and their peptide isomerases. Indeed, the above cases represent the most well-characterized peptide isomerases to date, with two of the three being the only known examples of identified eukaryotic peptide isomerases (15, 19).

As 14 DAACPs neuropeptides/peptide hormones have been identified in *Aplysia* (3, 4, 6, 12, 26, 27, 28, 29, 30), it is reasonable that one or more peptide isomerases exist within the same organism. In *Aplysia californica*, DAACPs display differential localization within the central nervous system (CNS). NdWFa (lowercase d represents the D-configuration for the following residue, lowercase a indicating C-terminal amidation) is localized to the abdominal ganglia, achatin-like neuropeptide precursor (ALNP)-derived DAACPs are localized to the pedal ganglia (6, 12, 31, 32, 33), and the pleurin precursor-derived peptides are located in the pleural, cerebral and buccal ganglia, although the later locations may be due to peptide transport (27, 29). In *Aplysia* DAACPs, the reported D-amino acids are located at the second or third residues from the N-termini (27), even though there is a wide range of amino acid residues at that location (34). As several G protein-coupled receptors in *A. californica* differentially recognize the all-L peptide form and the DAACP, and the cellular response changes between the all-L-peptide and the DAACP, this PTM is required for proper neuropeptide activity (3, 4, 6). As both the abdominal and pedal ganglia are expected to exhibit peptide isomerase activity based on the presence of endogenous DAACPs, we hypothesized that the L-form peptides NWFa, GFFD, and GYFD would be converted into DAACPs when incubated with abdominal and pedal ganglia tissue extracts.

In addition, a fascinating instance of novel peptide isomerase activity has been documented in the heart of *M. musculus*, although the observed conversion was low (35). In this case, the isomerase was active on a synthetic substrate that originated from platypus defensin-like-peptide 4 (DLP-4), with the sequence I(Nle)FdSdRdS, where norleucine was used as a redox inactive alternative to methionine. This discovery marked the first time that peptide isomerase activity was detected in a placental mammal, prompting us to investigate whether similar enzymes and potential endogenous peptide substrates also exist in *R. norvegicus* and *B. taurus*.

Here, we investigated peptide isomerase activity found in tissue extracts of *A. californica*, *R. norvegicus*, and *B. taurus*. In isolated extracts, isomerase activity was observed on substrates GFFD, GYFD, and NWFa for *A. californica*, substrates neuropeptide FF (NPFF) and I(Nle)FdSdRdS for *R. norvegicus*, and substrate NPFF for *B. taurus*. A study of the tissue specificity of the *R. norvegicus* peptide isomerase revealed enzyme activity across multiple organs and tissues, with the lungs displaying the highest activity overall. Intriguingly, some organs such as the liver had no isomerase activity. In *A. californica*, isomerase activity was found across the major CNS ganglia, with the highest activity in the pedal and cerebral ganglia. Other tissues such as the buccal mass and gills were absent of isomerase activity. We also investigated the effects of a variety of enzyme inhibitors on these isomerases, which hinted at key active site residues and structures needed for catalysis. Overall, this study provides a foundation for future investigations into the identity, cellular specificity, and functional significance of metazoan peptide isomerases, ultimately contributing to a deeper understanding of the biological impact of DAACPs.

## Results

### A tailored enzymatic assay for isomerase activity and LC-MS/MS-multiple reaction monitoring (MRM) analysis

Several techniques have been reported for the analysis of DAACPs (12, 26, 29, 31, 36). We initially utilized a tandem MS approach where the L- and D-peptide isomers were distinguished based on differential fragmentation patterns. While this approach worked well for a short peptide substrate (NWFa), the difference in fragmentation patterns between L- and D-peptide isomers is less pronounced for longer peptide sequences, making it harder to assign chirality based on ion ratios. Therefore, an LC-MS approach was developed in which the L- and D-peptide isomers elute from the column at different times because of their differences in structural configuration (34). In the analyses that are described herein the L/D assignment is based on matching the retention time of the sample peak to that of L/D-peptide standards.

**Figure 1:**
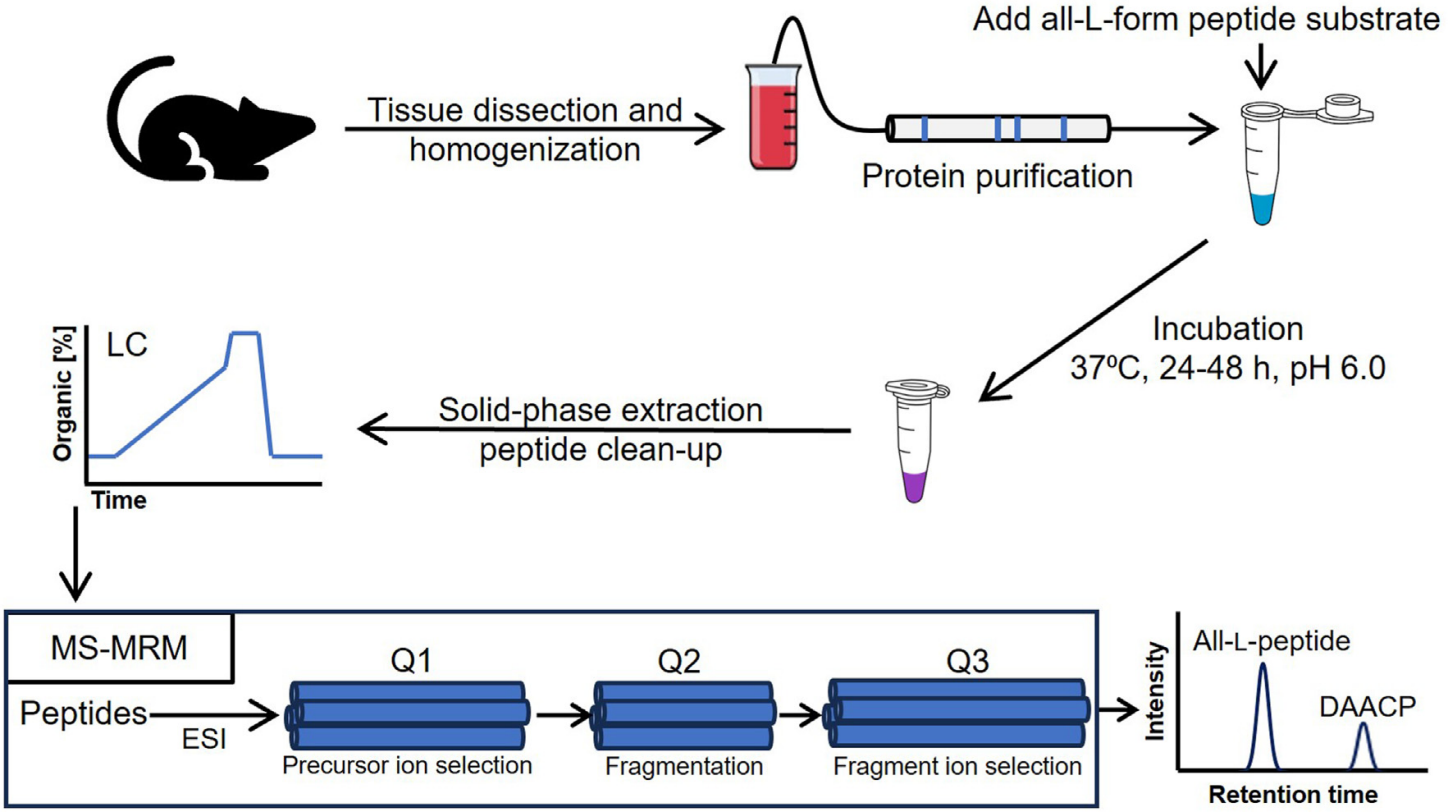
**Overview of the isomerase assay workflow**.

**Figure 2:**
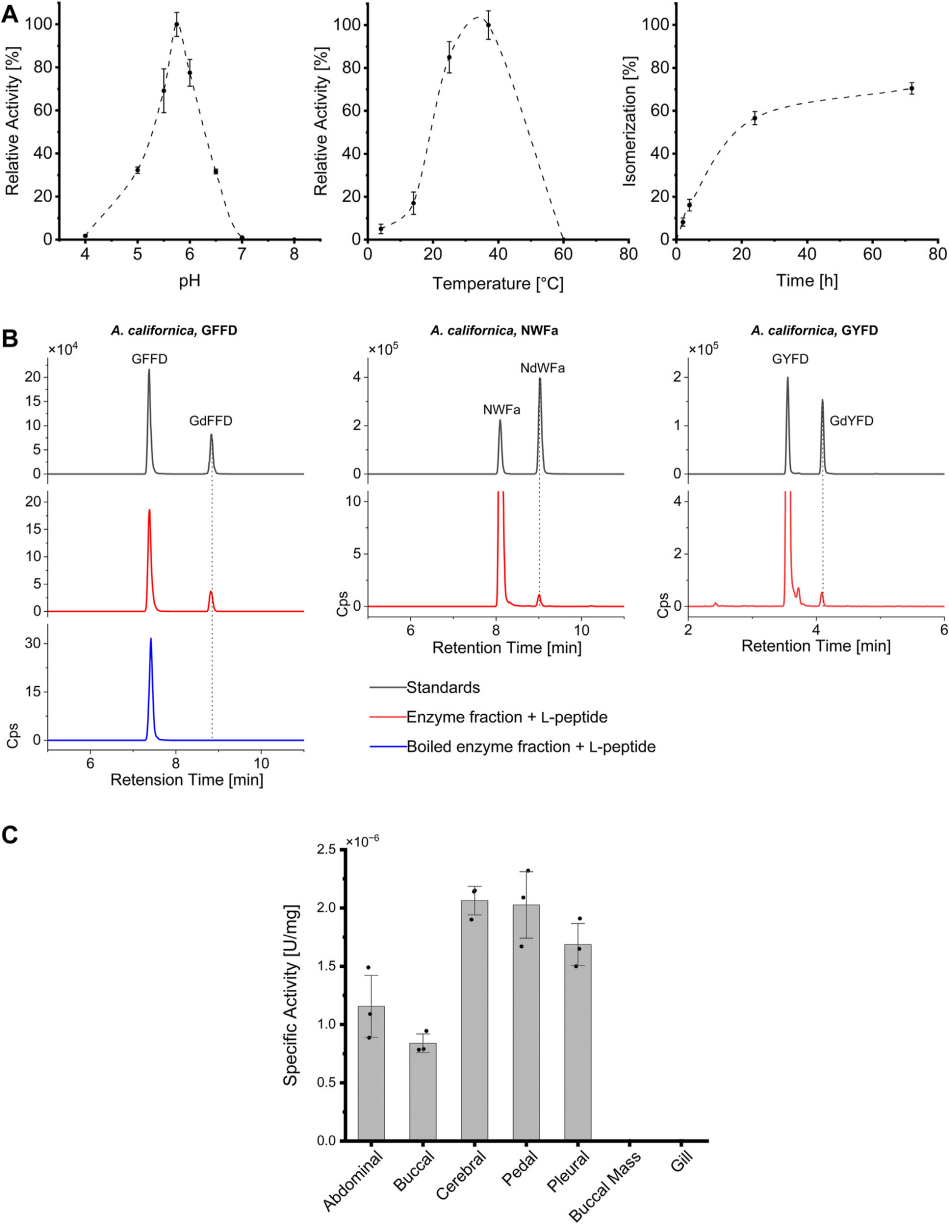
**Isomerase activity in*A. californica*.***A*, optimal pH, incubation temperature, and time course for the *A. californica* peptide isomerase. (*Left*) Assays performed at pH 4, 5, 5.5, 5.75, 6, 6.5, 7, and 8, at 37 °C for 24 h. (*Middle*) Temperature profile for isomerase activity. GFFD was incubated with an enzyme fraction at 4, 14, 25, 37, and 60 °C for 24 h. (*Right*) Time course of isomerase activity at 0, 2, 4, 24 and 72 h. GFFD was used as the standard substrate for all assays. Error bars represent standard deviation for N = 3 technical replicates for each condition assayed. *B*, conversion of GFFD, NWFa and GYFD in *A. californica* pedal ganglia extracts. *Black traces*: L/D standards were separated by retention time in LC/MS. Red traces: presence of isomerized product detected post-incubation, indicated by a *dotted line*. *Blue trace*: heat inactivated enzyme fraction. *C*, Isomerase-specific activity across *A. californica* tissues. Isomerase-specific activity was assessed across the major ganglia of the CNS, as well as in the buccal mass and gill. All ganglia were isomerase-active, while activity was absent in buccal mass and gill tissues. Individual points represent N = 3 biological replicates for each tissue, and error bars represent standard deviation.

Based on the enzymatic reaction conditions described for the frog, platypus, and spider isomerases (15, 19, 23), we developed a similar assay for isomerase activity (Fig. 1). In *A. californica*, the pH optimum was determined to be in the 5.8 to 6.1 range for this assay. This is interesting because the pH of organelles is critical for protein processing and trafficking and becomes progressively more acidic as the cargo moves through the secretory pathway. In our case, an acidic pH optimum is consistent with a neuropeptide processing enzyme found in the secretory pathway instead of a cytosolic enzyme that is active under neutral pH. The temperature optimum was found to be 37 °C, and isomerase activity was most observable after a 24 to 72 h incubation time (Fig. 2*A*).

### CNS extracts from *A. californica* contain an active peptide isomerase

Pedal ganglia extracts from *A. californica* were found to contain an active peptide isomerase, as demonstrated by the following experiment. To obtain an isomerase-active enzyme fraction, a total soluble protein homogenate was obtained from *A. californica* CNS tissue. This homogenate was then enriched in isomerase activity by chromatographic separation. GFFD was incubated in the enzyme fraction, and a peak in the GFFD MRM channel was observed at a retention time matching the GdFFD standard, indicating that GdFFD was detected post-incubation. The isomerase assay also verified the peptides NWFa and GYFD as substrates which could be converted to NdWFa and GdYFD. Control experiments ruled out the endogenous presence of GdFFD in the enzyme fraction and spontaneous L-to-D conversion through heat inactivation of the enzyme fraction (Fig. 2*B*). Moreover, the enzyme demonstrated the ability to convert GFFD both in the L-to-D direction and in the reverse D-to-L direction. This provides additional evidence supporting the enzymatic nature of the conversion (Fig. S1). Our results also suggest that the L-to-D reaction is faster than the D-to-L reaction, which was the case for all endogenous substrates studied in the frog, spider, and platypus L/D-peptide isomerases (15, 19, 23, 24).

The distribution of isomerase activity in the *A. californica* CNS as well as two peripheral tissues was assessed. Figure 2*C* shows that isomerase activity was detected in all major neural ganglia (buccal, cerebral, pedal, pleural, and abdominal ganglia) but not in the gill or buccal mass, which is the feeding apparatus in this animal. Previous characterizations of DAACPs in *A. californica* have shown that NdWFa is present in the abdominal ganglion (32), the ALNP-derived DAACPs GdFFD, GdYFD, and SdYADSKDEESNAALSDFA are mostly expressed in the pedal ganglia (3, 6, 12), and other DAACPs derived from the pleurin prohormone are present in the cerebral ganglia (27). Therefore, these results confirm the predicted presence of an isomerase in the abdominal, pedal, and cerebral ganglia, but isomerase activity in the buccal and pleural ganglia also hints at the existence of undiscovered DAACPs throughout the CNS and supports the hypothesis that L/D peptide isomerization is a widespread PTM in *A. californica* neuropeptides.

### Cofactor dependency and substrate specificity of the *A. californica* isomerase

Based on kinetic, isotope exchange, and planar substrate inhibition experiments, previously identified peptide isomerase enzymes are hypothesized to proceed *via* a two-base reaction mechanism, involving direct deprotonation and reprotonation of the α-carbon resulting in isomerization (17, 24, 37). To probe the enzymatic mechanism for the *A. californica* peptide isomerase, we explored the effects of various enzyme cofactors and peptide substrates on isomerase activity.

First, we observed that the addition of common cofactors utilized in proton transfer reactions did not affect the *A. californica* isomerase. Isomerase activity was not altered by 5 mM ethylenediaminetetraacetic acid (EDTA), which argues against the presence of divalent ions as cofactors. Addition of 2 mM MgCl_2_ or 4 mM ATP were tested because of their modulating effects on amino acid racemases (38, 39, 40), but neither was found to affect isomerase activity. Finally, the inability of 20 μM PLP or 1 mM AOAA (a PLP-inactivating reagent) (41, 42) to influence the isomerase reaction suggests that the enzyme operates independently of PLP as a cofactor (Fig. S2).

**Figure 3:**
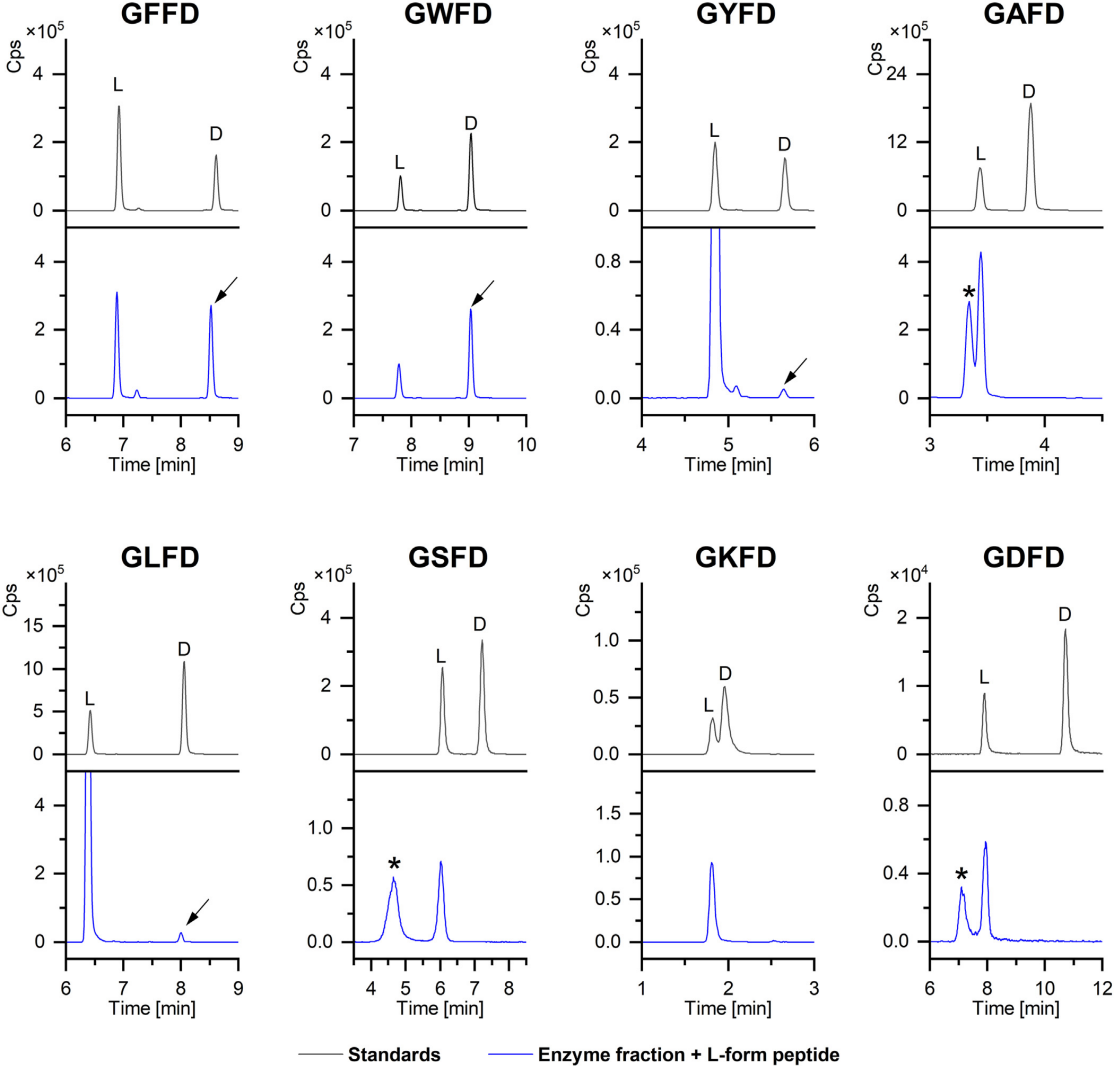
**Isomerization reactions for GFFD analogs.** Analogs contain different amino acid residues at position 2. Retention times of the L- and D-form standards for each peptide are shown (*black traces*), and *arrows* indicate the formation of the D-product when incubated with L-form (*blue traces*). The aromatic residues Phe, Tyr, and Trp were isomerized, along with the non-polar residue Leu. Unknown interference peaks are labeled (∗).

To gain further insight into the substrate requirements of the isomerase, we conducted an assay on a series of seven analogs of the standard substrate GFFD, each containing a single residue substitution at the second position. Our findings indicate that the isomerase can convert all three proteinogenic aromatic amino acid residues at the second position, namely, phenylalanine, tyrosine, and tryptophan. We also observed conversion for an L-leucine residue at position 2, while no isomerization was observed for the remaining GFFD analogs, containing alanine, serine, aspartate, and lysine at position 2 (Fig. 3). This specificity toward aromatic residues at position two is distinct from the frog isomerase, which has a low selectivity for residues at position 2, and all 13 amino acid residues tested were isomerized (20). On the other hand, the platypus isomerase was found to convert methionine, norleucine, and phenylalanine at position 2, out of the 11 amino acid residues that were tested (24). Further studies are needed to elucidate other substrate requirements (for example the effect of flanking residues in position 1 and 3) for the *A. californica* isomerase. If a consensus sequence is determined, it can also be used to screen a database of endogenous peptides to identify putative substrates for the isomerase, which are likely to exist as DAACPs.

Finally, three peptides were designed and synthesized to mimic the planar transition state molecule in a two-base mechanism. These peptides are analogs of the standard substrate GFFD and contain dehydrophenylalanine at the second position instead of phenylalanine. Two peptides were designed with a biotin handle that could be utilized for affinity purification if the analog peptide was found to be a potent inhibitor that was tightly bound to the isomerase. Isomerase assays were performed in the presence of each of the planar substrate analogs, and the results showed that GΔFFD (where ΔF represents dehydrophenylalanine) had a slight inhibition effect while the two longer analogs had no effect on isomerase activity (Fig. S3).

### *R. norvegicus* contains an active peptide isomerase

Although an endogenous DAACP has not been identified in mammals to date, a study by Koh *et al.* found evidence of peptide isomerase activity in the mouse heart, where a small fraction of the model substrate I(Nle)FdSdRdS was converted to I(dNle)FdSdRdS after incubation with tissue extracts (35). With our sensitive LC-MS/MS-MRM isomerase assay from the *A. californica* study, we also sought to explore the existence and activity of peptide isomerases in mammalian systems. First, we were able to detect isomerization of the same peptide substrate in mouse heart extracts using our assay, which corroborated the results from the previous study and provided further evidence of the existence of L-to-D peptide isomerization as a PTM in mammals (Fig. S4). Following this study, we applied our enzymatic assay procedure to *R. norvegicus* tissues, which offered an advantage in larger tissue size for enzyme extraction.

In addition to the model substrate I(Nle)FdSdRdS, we also identified NPFF as a potential peptide substrate for mammalian peptide isomerases. Mammalian NPFF (sequence FLFQPQRFa) is an FMRFamide-related peptide implicated in various physiological functions, including pain and analgesia control through interactions with the opioid system (43, 44, 45). Several reasons suggest NPFF may exist as a DAACP in mammals. For example, *Mytilus*-FFRFamide (AdLAGDHFFRFa), another peptide from the FMRFamide-related peptide family, contains a D-leucine in the second position and is known to be an endogenous and bioactive DAACP from the bivalve mollusk *M. edulis* (46). Moreover, NPFF was isomerized when incubated with the frog L/D-peptide isomerase in a study by Jilek *et al.* (20).

**Figure 4:**
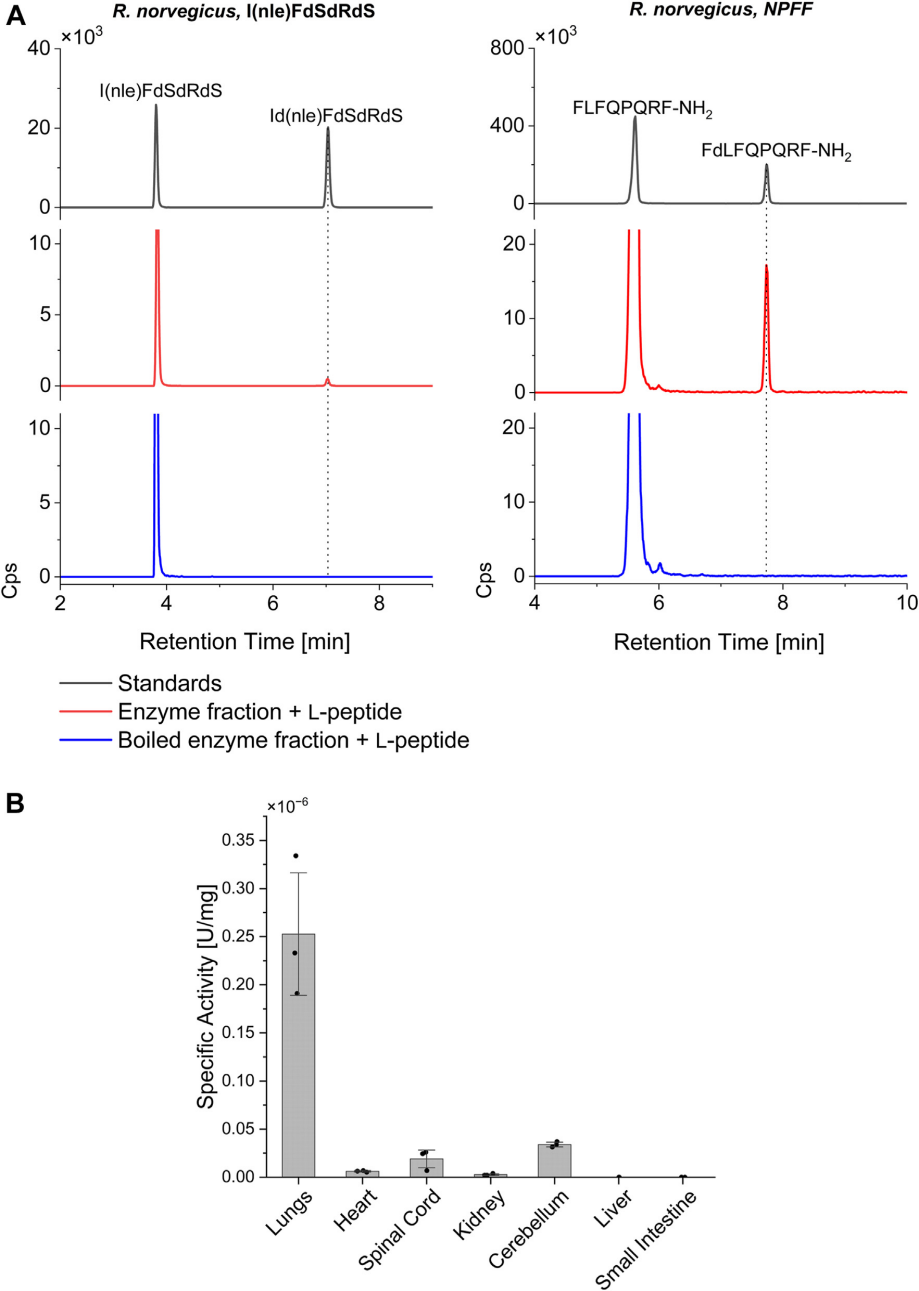
**Isomerase activity in*R. norvegicus*.***A*, *R. norvegicus* isomerase activity on NPFF (Sequence: FLFQPQRF-NH_2_) and I(Nle)FdSdRdS. All enzyme fractions were obtained from lung tissue, and two peptides were evaluated as substrates. In each panel, the *black traces* show the retention times of synthetic L- and D-form peptide standards, *red traces* represent each L-substrate incubated with the isomerase, and *blue traces* represent heat-treated fractions. The retention time of the D-form product is indicated with a *dotted line*, showing the conversion of both substrates. *B*, *R. norvegicus* isomerase activity across tissues. Specific activity is shown across seven tissue regions, using NPFF as substrate. Specific activity was highest in the lungs, while the liver and small intestine were absent in isomerase activity. N = 3 biological replicates for each tissue, and error bars represent standard deviation.

Via the LC-MS/MS-MRM method, we showed that NPFF was isomerized from FLFQPQRFa to FdLFQPQRFa when incubated with enzyme extracts from *R. norvegicus* prepared *via* centrifugal ultrafiltration, indicating its potential as an endogenous substrate for the mammalian peptide isomerase. The model substrate I(Nle)FdSdRdS was also found to be isomerized into I(dNle)FdSdRdS under the same incubation condition. Additionally, heat treatment of the enzyme fraction prior to incubation with either substrate prevented L-to-D conversion, providing further evidence for the enzymatic nature of the conversion (Fig. 4*A*).

To highlight the distribution of isomerase activity between CNS tissues and visceral organs, two regions from the rat central nervous system (cerebellum and spinal cord), as well as five viscera (lung, heart, liver, kidney, and small intestine), were screened for isomerase activity on NPFF (Fig. 4*B*). Notably, the lungs exhibited the highest specific activity compared to all the other tissues tested. Among the remaining tissues displaying isomerase activity, the cerebellum, spinal cord, heart, and kidney showed progressively decreasing specific activities, with the cerebellum having the second highest and the kidney having the lowest. While the formation of the D-form peptide was not observed in the liver or small intestine, elimination of isomerase activity due to protease degradation was ruled out after incubation with a custom cocktail of protease inhibitors, showing only the presence of the l-form peptide substrate.

### *B. taurus* contains an active peptide isomerase

**Figure 5:**
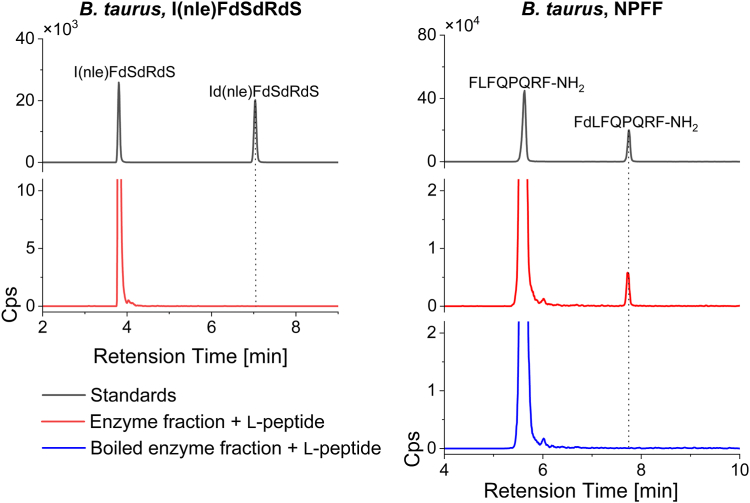
***B. taurus* isomerase activity on NPFF (Sequence: FLFQPQRF-NH**_**2**_**).** All enzyme fractions were obtained from lung tissue, and two peptides were evaluated as substrates. In each panel, the *black traces* show the retention times of synthetic L- and D-form peptide standards; *red traces* represent each L-substrate incubated with the isomerase. The retention time of the D-form product is indicated with a *dotted line*, showing conversion of NPFF, but not I(Nle)FdSdRdS.

To demonstrate whether the isomerase activity is unique to rodents or found in other mammals, as well as to locate a model better suited for future large-scale isomerase purification efforts, cow lung tissue protein fractionate prepared *via* centrifugal ultrafiltration was subjected to the enzyme preparation and analysis protocol. Our analysis shows that NPFF was readily converted while I(Nle)FdSdRdS did not display observable conversion (Fig. 5). Moreover, when the enzyme fraction was subjected to heat treatment before incubation with NPFF, the L-to-D conversion was prevented. This additional evidence strongly supports the enzymatic nature of the conversion process. Notably, this discovery represents the first observation of such L/D peptide isomerase activity in cows, providing further evidence for the presence of peptide isomerase activity across mammals.

### Inhibitory profile of the *A. californica, R. norvegicus, and B. taurus* peptide isomerases

**Figure 6:**
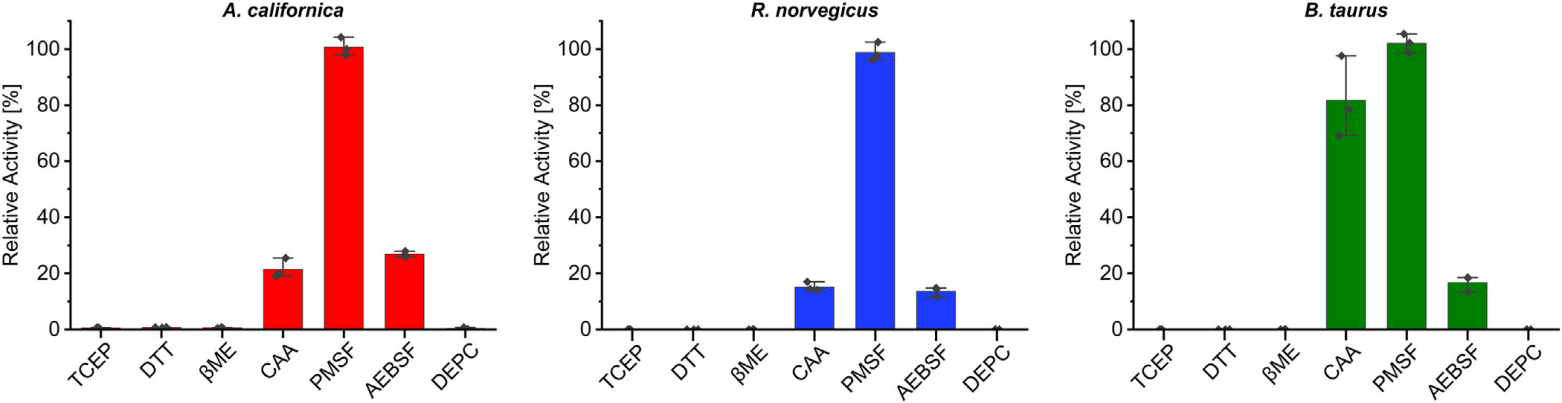
**Inhibitory profile *A. californica, R. norvegicus*, and *B. taurus* isomerases.** Isomerase activity was absent when incubated with 100 mM DTT, 5% BME, 50 mM TCEP and 0.1% DEPC. 1 mM AEBSF caused a decrease in relative activity, while 1 mM PMSF did not show an effect. While the *A. californica* and *R. norvegicus* isomerases were susceptible to 20 mM CAA, *B. taurus* isomerase activity appeared resistant to cysteine alkylation. Individual points represent N = 3 technical replicates for each tissue, and error bars represent standard deviation. Activity is relative to positive controls for each enzyme.

Considering the susceptibility of the peptide isomerase from *A. aperta* to the serine protease inhibitor phenylmethylsulfonyl fluoride (PMSF) (16), we investigated the possibility of a similar inhibitory effect. Surprisingly, 1 mM PMSF had no impact on peptide isomerization, whereas 1 mM (2-aminoethyl) benzenesulfonyl fluoride (AEBSF) exhibited a distinct inhibitory effect on all peptide isomerases. Moreover, due to the resemblance between the *A. aperta* peptide isomerase and serine proteases, which commonly possess a Ser-His-Asp catalytic triad (47), we investigated the effect of diethylpyrocarbonate (DEPC) on isomerase activity. Our findings revealed that 0.1% DEPC displayed an even stronger inhibitory effect, likely attributed to its selective modification of His residues. These results share similarity to the frog isomerase, which was not inhibited by PMSF but was found to be sensitive to DEPC and hypothesized to contain a histidine residue in the active site (19). Cysteine also appears to play a crucial role in isomerase activity, as the reducing agents tris(2-carboxyethyl)phosphine (TCEP), β-mercaptoethanol (BME), and dithiothreitol (DTT) completely inhibit activity at concentrations of 50 mM, 5%, and 100 mM, respectively. While 20 mM chloroacetamide (CAA), a cysteine alkylating reagent, greatly reduces substrate conversion in *A. californica* and *R. norvegicus* peptide isomerases, its effect on the *B. taurus* isomerase is attenuated (Fig. 6).

## Discussion

### Shared attributes of the *A. californica*, *R. Norvegicus*, and *B. Taurus* peptide isomerases

The novel eukaryotic peptide isomerase activities presented here, combined with others reported previously (15, 19, 22, 35, 48, 49), illustrate the broad phylogenetic prevalence of this class of enzymes. Indeed, it is a great indication of biological significance if a specific function, structure, or mechanism is present across a wide range of species. Furthermore, the localization of isomerase activity away from venom or skin secretions expands the functional significance of peptide isomerization beyond toxins.

The L-to-D isomerization of a specific residue within a peptide is a key function of eukaryotic peptide isomerases, and the conversion of the DAACPs listed above per each peptide isomerase above illustrates this point well. Additionally, our investigations into the inhibitory profiles of these enzymes suggest potential similarities in the catalytic residues involved.

Given the similarity between the *A. aperta* peptide isomerase and serine proteases, which commonly feature the Ser-His-Asp catalytic triad (47), it is plausible that the *A. californica*, *R. norvegicus*, and *B. taurus* enzymes share a similar active site configuration. We provide support for this hypothesis by exhibiting their shared susceptibility to irreversible covalent modification of nucleophilic residues through AEBSF and their shared vulnerability to histidine alkylation *via* DEPC. However, we must acknowledge the potential reactive promiscuousness of AEBSF and DEPC to other nucleophilic residues, such as Cys and Thr (50, 51).

In contrast to the enzymes isolated from *Bombina variegata* and *Ornithorhynchus anatinus* (19, 24), the *A. californica* and *R. norvegicus* enzymes are susceptible to cysteine alkylation, indicating the involvement of an active site –SH group over an active site –OH group. Interestingly, the isomerase from *B. taurus* appears to be less susceptible to cysteine alkylation, which indicates an active site –OH group.

Additionally, it was previously reported that the enzymes isolated from *B. variegata* and *O. anatinus* were not susceptible to PMSF, and thus not likely to contain a serine-protease-like reactive triad (19, 24). Given that the *A. californica*, *R. norvegicus*, and *B. taurus* enzymes were susceptible to AEBSF rather than PMSF, it would be intriguing to investigate whether the enzymes from *B. variegata* and *O. anatinus* share this susceptibility, providing further evidence for the serine-protease-like reactive triad as a possible feature of peptide isomerase enzymes.

Finally, the complete inhibition of all three isomerases upon the disruption of disulfide bonding, coupled with their functional pH range under assay conditions, provides additional evidence supporting the notion that these enzymes operate along the secretory pathway (52, 53).

### Differences between the *A. californica*, *R. Norvegicus*, and *B. Taurus* peptide isomerases

The analysis of substrate preference between the peptide isomerases illustrates the first key difference. We demonstrated *A. californica* peptide isomerase activity on the substrate GFFD, while the *R. norvegicus* and *B. taurus* peptide isomerases showed differential levels of activity on the substrates NPFF and I(Nle)FdSdRdS. While the differences between substrate preference between mollusks and mammals are expected, the preference of NPFF in *B. taurus* highlights a dissimilarity between the mammalian peptide isomerases. A possible reason for this difference lies in the endogenous nature of NPFF, compared to the synthetic I(Nle)FdSdRdS. Moreover, the amount of converted NPFF in *B. taurus* compared to *R. norvegicus* appears to be lower. While this may be indicative of a difference in active site structure, it may also reflect the tissue isolation procedure. Indeed, it is much easier to test the entire organ in a series of assays for rats than for cows.

Secondly, while multi-day incubation time frames are shared by these isomerases, the maximum conversion of substrate differs between the *A. californica* and *R. norvegicus* isomerases, with the *A. californica* isomerase showing a higher percent conversion of substrate per unit time (Fig. S5, *A* and *B*). While this may reflect an inherent difference due to active site residues or expression levels, it may also reflect NPFF as an unideal substrate for the mammalian isomerase, given that it has not yet been confirmed to exist endogenously in DAACP form.

Finally, while tissue-specific activity investigations revealed the presence of isomerase activity in the CNS of *A. californica* predominantly, the mammalian isomerase is most functional in the lungs, while displaying lesser activity in parts of the CNS.

## Conclusion

Overall, our findings highlight the importance of peptide isomerases in biological systems and their potential implications for understanding the cellular specificity and functional significance of DAACPs. We have gained valuable insights into the presence and activity of these enzymes by examining different organisms and tissues. Moving forward, our assay can serve as a solid foundation to identify the endogenous isomerase enzymes. Additionally, our study has opened new avenues for exploring the presence and activity of these enzymes in other organisms.

We speculate that several orphan mammalian GPCRs, currently being tested using libraries of prohormone-derived all L-amino acid-containing peptides, may require a DAACP, resulting in missed substrate-receptor interactions. Identifying the isomerase would enable further studies on its colocalization with known prohormones. Moreover, the isomerase activity observed in the lungs suggests the presence of distinct functions, such as peptides that contribute to the innate immune response and exhibit antimicrobial activity, similar to known bacterial peptides with D-amino acids.

## Experimental procedures

Detailed procedures can be found in the Supporting Information.

### Isomerase assay using LC-MS/MS-MRM

#### *A. californica* enzyme preparation

*A. californica* (100–200 g) were obtained from the University of Miami/NIH National Resource for Aplysia and housed in an aquarium with 14 °C aerated and filtered artificial seawater (Instant Ocean, Aquarium Systems Inc.) Animals were used within 1 week of arrival. Prior to dissection, animals were anesthetized by an injection of 366 mM MgCl_2_ at 50% (v/w) body weight into the body cavity. Neural ganglia and other tissues were dissected from each animal and stored at −80 °C until used. Frozen ganglia were reduced to a coarse powder in a stainless-steel tissue pulverizer that was pre-chilled on dry ice. Refrigerated 1× PBS with 5 mM EDTA was added at a ratio of 10 ml per gram of pulverized tissue. The mixture was then homogenized on ice using a PowerGen 125 Homogenizer (Fisher Scientific) with a 7 mm × 115 mm probe for five bursts of 5 s each. The homogenate was centrifuged at 10,000*g* for 10 min. The supernatant was filtered through a 0.45 μm syringe filter and adjusted to a 1 M ammonium sulfate solution by dropwise addition of saturated (4 M) ammonium sulfate, left on ice for 20 min, then centrifuged at 12,500*g* for 10 min. The supernatant was then fractionated on an AKTA fast protein liquid chromatography system (GE Biosciences) with hydrophobic interaction chromatography on a Phenyl-Superose HR 5/5 column (GE Biosciences) at a flow rate of 0.5 ml/min. The buffer was 25 mM Tris-HCl pH 8.3 with 5 mM EDTA, and the gradient was 1 M to 0 M ammonium sulfate over 15 column volumes. 40 fractions were collected at 0.5 ml/Fr; each fraction was tested for isomerase activity and active fractions (typically around fraction #25–30) were used as the enzyme sample in subsequent assays.

#### *A. californica* isomerase assay

For each assay, 50 μl of enzyme sample was buffer exchanged into 30 μl of 50 mM phosphate buffer with 5 mM EDTA, pH 6 (this is the standard assay buffer) using a Nanosep Centrifugal Device with the Omega 10 kDa MWCO membrane (Pall). The samples for heat treatment experiments were placed in boiling water for 10 min prior to incubation. Enzyme samples were incubated with 10 μM peptide substrate at 37 °C for 24 h unless otherwise stated.

#### *R. norvegicus/B.taurus* enzyme preparation

Male Sprague-Dawley rats (1–3 months old) were obtained from Charles River. Work with the rodents was performed in accordance with an animal use protocol approved by the Institutional Animal Care and Use Committee at the University of Illinois at Urbana-Champaign and in accordance with local and federal regulations. Cow lung samples were collected from a single animal after the standard slaughtering procedure performed in The Meat Science Laboratory at the College of Agricultural, Consumer and Environmental Sciences, University of Illinois at Urbana-Champaign. Collected tissues were frozen on dry ice immediately and stored at −80 °C until use.

Frozen tissue was reduced to a coarse powder in a stainless-steel tissue pulverizer that was pre-chilled on dry ice. Refrigerated 50 mM citrate-phosphate buffer with 5 mM EDTA was added at a ratio of 10 ml per gram of pulverized tissue. The mixture was then homogenized on ice using a PowerGen 125 Homogenizer (Fisher Scientific) with a 7 mm × 115 mm probe for five bursts of 5 s each. The homogenate was centrifuged at 10,000*g* for 10 min. After centrifugation, the solid material was discarded, and the supernatant was filtered through Corning surfactant-free cellulose acetate (SFCA) 0.45 μm syringe filters. This homogenate was either utilized directly for enzymatic assays or subjected to further fractionation *via* molecular weight cut-off filtration: Protein homogenate was added to an Amicon Ultra-15 Centrifugal Filter Unit with 100 kDa MWCO (Millipore Sigma). The protein solution was passed through the filter by a centrifugal force of 7200*g* for 40 min. The resultant filtrate was then passed through an Amicon Ultra-5 Centrifugal Filter Unit with 50 kDa MWCO (Millipore Sigma), run for 5 min at 3000*g*. The resultant filtrate was then used for isomerase activity assays. Additionally, isomerase activity was lost in the filtrate of a 30 kDa MWCO filter, and enhanced degradation was evident in the filtrate of a 100 kDa MWCO filter (Fig. S6).

#### *R. norvegicus/B.taurus* isomerase assay

For the investigations on tissue-specific activity, 100 μl of enzyme sample was concentrated and exchanged into 30 μl of 50 mM citrate-phosphate buffer with 5 mM EDTA, pH six using a Nanosep Centrifugal Device with the Omega 10-kDa MWCO membrane (Pall). The protease inhibitors leupeptin, aprotinin, and E−64 were added from a 20× stock of the combined protease inhibitors to a concentrated and buffer-exchanged enzyme fractionate. The working concentration of the inhibitors was 50× relative to the standard working concentration of the Halt Protease Inhibitor Cocktail (ThermoFisher Scientific). The resultant solution was adjusted to a volume of 55 μl by adding 50 mM citrate-phosphate buffer with 5 mM EDTA, pH 5.9, and L-NPFF (FLFQPQRFa) to a concentration of 2 μM. Prior to incubation, the samples for heat treatment experiments were placed in boiling water for 10 min. The isomerase reaction was then induced by leaving the sample for 48 h at 37 °C.

#### LC-MS/MS-MRM sample preparation

All samples were desalted and concentrated with solid-phase extraction using reversed-phase C18 ZipTip pipette tips (Millipore) prior to LC-MS analysis. Briefly, the sample was adjusted to 0.1% trifuoroacetic acid (TFA), pH < 4; the ZipTip was washed twice with 10 μl ACN and twice with 10 μl 0.1% TFA in water, then the sample was loaded onto the resin by aspirating and dispensing the sample 10 times through the tip. The tip was then washed three times with 10 μl of 5% methanol in water with 0.1% TFA, to remove salts and unbound molecules. The purified peptide sample was eluted in 20 μl of 50% ACN in water with 0.1% FA.

#### LC-MS/MS-MRM analysis

Prepared isomerase assay samples were analyzed in the MRM mode on an LC-MS/MS system that consisted of an Advance ultra-high performance LC coupled to an EVOQ triple quadrupole mass spectrometer (Bruker). The MS was operated with the following parameters: heated electrospray ionization (HESI) source, (±) spray voltage = 4000 V, cone temperature = 250 °C, cone gas flow = 20, heated probe temperature = 400 °C, probe gas flow= 45, and nebulizer gas flow = 50. The LC column was an XSelect CSH C18 3.5 μm column, 4.6 × 150 mm (Waters), and the solvents were water with 0.1% FA (solvent A) and ACN with 0.1% FA (solvent B). The 15 min gradient was 5 to 15% B in 2 min, 15 to 30% B in 8 min, 30 to 70% B in 1 min, 70% B for 1 min, 70- 5% B in 1 min, and 5% B for 2 min, at a flow rate of 1 ml/min.

#### Enzyme activity calculations

To calculate the enzyme relative activity, the following formula was used: Relative Activity (%) = (Experimental enzyme activity/Reference enzyme activity) × 100. The reference condition utilized in this study was the enzyme's activity in the absence of any experimental manipulation (*i.e.*, the positive control condition). To determine enzyme-specific activity, the protein content of the enzyme sample was determined using a standard BCA protein assay. Subsequently, the D-peptide peak area obtained *via* the LC-MS/MS-MRM method was obtained to calculate the concentration of substrate converted, which was then divided by total incubation time to obtain enzyme activity in units. By dividing the enzyme activity by the protein content, we obtained the enzyme-specific activity, which is expressed as units of enzyme activity per mg of protein (*e.g.*, units/mg protein).

## Data availability

Detailed information on the experimental design, sample preparation, and data analysis, as well as any raw data files, including chromatograms, are available on request from the corresponding author: jsweedle@illinois.edu.

## Supporting information

This article contains supporting information including an expanded methods section and six figures.

## Conflict of interest

The authors declare that they have no known competing financial interests or personal relationships that could have appeared to influence the work reported in this paper.
