# Supplementary material for: A novel series of metazoan L/D peptide isomerases
Source: J Biol Chem. 2024 Jun 8;300(7):107458. doi: 10.1016/j.jbc.2024.107458 (PMC11277431; doi:10.1016/j.jbc.2024.107458)
Supplement: Supporting information [file mmc1.docx]

**Supporting Information**

**A novel series of metazoan L/D peptide isomerases: analysis and partial characterization**

Harvey M. Andersen ^1,3,4, ¥^, Hua-Chia Tai ^1,3, ¥,∞^, Stanislav S. Rubakhin^1,2^, Peter M. Yau,^5^ Jonathan V. Sweedler^1,2,3,4*^

^1^Beckman Institute, University of Illinois, Urbana-Champaign, Urbana, IL 61801

^2^Department of Chemistry, University of Illinois, Urbana-Champaign, Urbana, IL 61801

^3^Department of Molecular and Integrative Physiology, University of Illinois, Urbana-Champaign, Urbana IL 61801

^4^Institute for Genomic Biology, University of Illinois, Urbana-Champaign, Urbana, IL 61801

^5^Department of Cell and Developmental Biology, University of Illinois, Urbana-Champaign, Urbana IL 61801

^¥^These authors contributed equally to this work.

^∞^ Current address: Chemical Process Development, Bristol Myers Squibb Company, 1 Squibb Drive, New Brunswick, NJ 08903, USA

^*^Corresponding author

**Table of Contents**

**Supporting Materials and Methods…...…………………………….………………..….S-3**

**Supporting Tables and Figures…………...…………………………….……….…........S-6**

Table S1. Activity comparison in total protein homogenate from lung tissue…........….S-6

Figure S1. Reversibility of the *A. californica* isomerase………………………………….S-7

Figure S2. Effect of cofactors on *A. californica* isomerase activity…………….………..S-8

Figure S3. Effect of synthetic planar substrate analogs on isomerase activity…..…....S-9

Figure S4. Isomerase activity in the mouse heart…………………..………………..….S-10

Figure S5. Reaction time course comparison between *A. californica* and *R. norvegicus* peptide isomerases………………………………………………………………….....…..S-11

Figure S6. Isomerase activity after centrifugal filtration…………………………………S-12

**Supporting Materials and Methods**

**Materials.** Unless otherwise specified, all solvents and organic chemicals were purchased from Sigma-Aldrich. The peptides GFFD, and GdFFD were synthesized by the Protein Sciences Facility of the Roy J. Carver Biotechnology Center at the University of Illinois at Urbana-Champaign; GYFD and GdYFD were synthesized by CPC Scientific; GAFD, GLFD, GWFD, GSFD, GKFD, GDFD, and GdSFD were synthesized by Genscript; GdAFD, GdLFD, GdWFD, GdKFD, and GdDFD were synthesized by solid-phase peptide synthesis as described (3). The peptides I(Nle)FdSdRdS, I(dNle)FdSdRdS, and FdLFPQRFa were synthesized by Genscript; and Neuropeptide FF (FLFPQRFa) was purchased from Genscript. LC-MS grade water, acetonitrile (ACN), and formic acid (FA) were purchased from Thermo Fisher Scientific**.** Protected amino acids were purchased from Novabiochem, Chem Impex, and AnaSpec. Benzotriazole-1- yl-oxy-tris-pyrrolidino-phosphonium hexafluorophosphate (PyBOP) was purchased from Novabiochem. N-Hydroxybenzotriazole (HOBt) was purchased from AnaSpec. Wang resins preloaded with the C-terminal residue were purchased from Novabiochem and AnaSpec. NovaPEG Rink amide resin was purchased from Novabiochem.

**Standard Homogenization Procedure.** Frozen tissue was reduced to a coarse powder in a stainless-steel tissue pulverizer that was pre-chilled in on dry ice. Refrigerated 1X PBS with 5 mM EDTA was added at a ratio of 10 mL per gram of pulverized tissue. The mixture was then homogenized on ice using a PowerGen 125 Homogenizer (Fisher Scientific) with a 7mm x 115mm probe for 5 bursts of 5 s each. The homogenate was centrifuged at 10,000 x g for 10 min. After centrifugation, the solid material was discarded, and the supernatant was filtered through Corning® surfactant-free cellulose acetate (SFCA) 0.45 μm syringe filters to obtain the clarified homogenate.

***R. norvegicus* protein homogenate preparation and isomerase assay (Table S1).** Frozen lung tissue was taken through the standard homogenization procedure with the following changes: homogenization buffer additives varied between +/- 20% glycerol or +/- 0.5% Triton X-100, with the following buffers: 1X PBS +5 mM EDTA, pH 7.2, 50 mM Tris + 5 mM EDTA, pH 8.0 and 50 mM citrate-phosphate + 5 mM EDTA, pH 5.9.

For each assay, 100 μL of enzyme sample was concentrated and exchanged into 30 μL of 50 mM phosphate or citrate-phosphate buffer with 5 mM EDTA, pH 6 using a Nanosep Centrifugal Device with the Omega 10 kDa MWCO membrane (Pall). E-64, Aprotinin and Leupeptin protease inhibitors were added to a final concentration of 750 μM, 40 μM and 1 mM respectively. NPFF peptide substrate was added to the enzyme sample to a final concentration of 2 μM. All samples were incubated at 37 °C for 48 h. Samples were desalted and concentrated prior to LCMS analysis (standard procedure).

***M. musculus* enzyme preparation and isomerase assay (Figure S4).** Frozen lung tissue was taken through the standard homogenization procedure. The resulting homogenate was adjusted to a 1 M ammonium sulfate solution by drop wise addition of saturated (4 M) ammonium sulfate, left on ice for 20 min, then centrifuged at 12,500 x g for 10 min. The supernatant was then fractionated on an AKTA fast protein liquid chromatography (FPLC) system (GE Biosciences) with hydrophobic interaction chromatography on a Phenyl-Superose HR 5/5 column (GE Biosciences) at a flow rate of 0.5 mL/min. The buffer was 25 mM Tris-HCl pH 8.3 with 5 mM EDTA, and the gradient was 1 M to 0 M ammonium sulfate over 15 column volumes. 40 fractions were collected at 0.5 mL/Fr; each fraction was tested for isomerase activity and isomerase activity was found between fractions 32-36. The active fractions were used as the enzyme sample in subsequent assays.

For each assay, 100 μL of enzyme sample was concentrated and exchanged into 30 μL of 50 mM phosphate with 5 mM EDTA, pH 6 using a Nanosep Centrifugal Device with the Omega 10 kDa MWCO membrane (Pall). 2 μL of 100 μM I(nle)FdSdRdS peptide substrate was added to the enzyme samples. All samples were incubated at 37 °C for 48 h. Samples were desalted and concentrated prior to LCMS analysis (standard procedure).

***R. norvegicus/A. californica* enzyme preparation and isomerase assay (Figures S5, S6).** Frozen lung tissue was taken through the standard homogenization procedure with refrigerated 50mM citrate-phosphate + 5 mM EDTA, pH 6. Protein homogenate from *R. norvegicus* or *A. californica* was added to an Amicon® Ultra-15 Centrifugal Filter Unit with 100kDa MWCO (Millipore Sigma). The protein solution was passed through the filter by a centrifugal force of 7,200 x g for 40 min. 100 μL of this filtrate was saved for an enzyme assay. The remaining filtrate was then passed through an Amicon® Ultra-5 Centrifugal Filter Unit with 50kDa MWCO (Millipore Sigma), run for 5 min at 3000 x g. 100 μL of this filtrate was saved for an enzyme assay. Finally, the remaining filtrate was passed through an Amicon® Ultra-5 Centrifugal Filter Unit with 30kDa MWCO (Millipore Sigma), run for 5 min at 3000 x g. 100 μL of this filtrate was saved for an enzyme assay.

For each assay, 100 μL of enzyme sample was concentrated and exchanged into 30 μL of 50 mM citrate-phosphate with 5 mM EDTA, pH 6 using a Nanosep Centrifugal Device with the Omega 10 kDa MWCO membrane (Pall). E-64, Aprotinin and Leupeptin protease inhibitors were added to a final concentration of 750 μM, 40 μM and 1 mM respectively. NPFF peptide substrate was added to the enzyme sample to a final concentration of 2 μM. All samples were incubated at 37 °C for 48 h. Samples were desalted and concentrated prior to LCMS analysis (standard procedure)

For each assay, 100 μL of enzyme sample was concentrated and exchanged into 30 μL of 50 mM phosphate with 5 mM EDTA, pH 6 using a Nanosep Centrifugal Device with the Omega 10 kDa MWCO membrane (Pall).

For figure S5, NPFF peptide substrate was added to the *R. norvegicus* enzyme concentrate to a final concentration of 2 μM and GFFD peptide substrate was added to the *A. californica* enzyme concentrate to a final concentration of 10 μM. All samples were incubated at 37 °C for 1-5 d. Samples were desalted and concentrated prior to LCMS analysis (procedure in main text).

For figure S6 I(Nle)FdSdRdS peptide substrate was added to the enzyme concentrate to a final concentration of 2 μM. All samples were incubated at 37 °C 48 h. Samples were desalted and concentrated prior to LCMS analysis (procedure in main text).

**Planar substrate analogs*.*** The peptides GΔFFD-OH, GΔAFDSKDY-NH_2_, and GΔFFDSKDEKdAdY-NH_2_ were designed and synthesized s by Dr. James Checco. All three peptides contain a dehydro-amino acid residue at the second position; GΔAFDSKDY-NH_2_ and GΔFFDSKDEKdAdY-NH_2_ contain an ε-biotinylated lysine as a molecular handle; and GΔFFDSKDEKdAdY-NH_2_ was designed with D-amino acids at the C-terminal to increase resistance to carboxypeptidase.

Solid-phase peptide synthesis was based on Fmoc protection of the main chain amine. Peptides with a C-terminal acid were synthesized on the solid phase using Wang resin preloaded with the C-terminal residue (Novabiochem or Anaspec). Peptides with a C-terminal amide were synthesized on NovaPEG Rink Amide resin (Novabiochem). Coupling reactions were performed by treating the resin with a solution of ≥4 molar equivalents of Fmoc-protected amino acid with appropriate side chain protecting groups, activated with benzotriazole-1-yl-oxy-tris-pyrrolidino-phosphonium hexafluorophosphate (PyBOP) and N,N-diisopropylethylamine (DIEA) (1:1:2 amino acid:PyBOP:DIEA molar ratio) in a solution of 0.1 M N-hydroxybenzotriazole in N-methyl-2-pyrrolidone. Coupling reactions were allowed to proceed at room temperature (RT) for >40 min with stirring or gentle shaking, after which the resin was rinsed with three to five washes of dimethylformamide (DMF). Deprotection of the Fmoc protecting group was performed in a solution of 20% piperidine in DMF for 20 min at RT with gentle stirring or shaking, after which the resin was rinsed with three to five washes of DMF.

After the completion of the synthesis, peptides were cleaved from the resin and side chain protecting groups were removed using a solution of 95% trifluoroacetic acid, 2.5% H2O, and 2.5% triisopropylsilane for >3 h. After cleavage, most of the cleavage solution was removed by evaporation, and the peptides were dissolved in a water/acetonitrile (ACN) mixture or dimethyl sulfoxide for high performance liquid chromatography (HPLC) purification.

**Table S1: Activity comparison in total protein homogenate from *R. norvegicus* lung tissue.** Isomerase activity is highly dependent on the pH of the homogenization buffer. Two assay buffers at pH 7.4 (Phosphate Assay Buffer) and pH 5.9 (Citrate-Phosphate Buffer) are also compared. Activity is higher in all buffers that contain citrate. In homogenates that included protease inhibitors, the inhibitors used were Aprotinin, Leupeptin, and E-64, at the working concentration of the Halt Protease Inhibitor Cocktail (Thermo Fisher Scientific). No difference in activity was seen with homogenates that included glycerol, while a slight increase in activity was seen when a mild detergent was added.

**
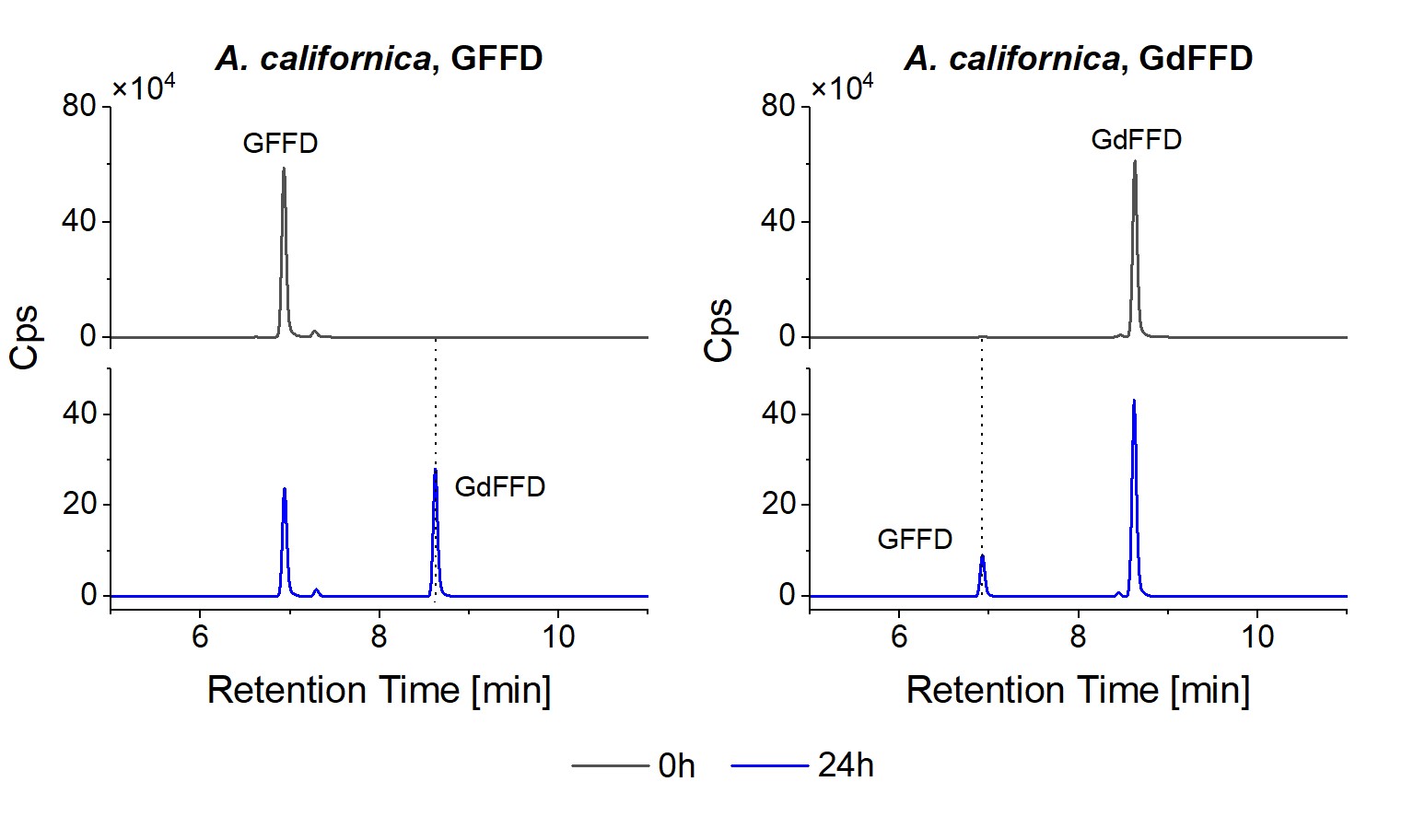
**

**Figure S1. Reversibility of the *A. californica* isomerase.** (left) Incubation with GFFD. (right) Incubation with GdFFD. Samples were analyzed at 0 h (top black traces) and 24 h (bottom blue traces). Dotted lines refer to the expected retention time of the product.


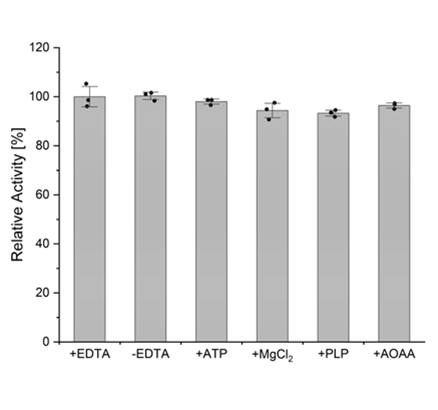


**Figure S2: Cofactor dependency of the *A. californica* isomerase.** GFFD was incubated with isomerase fractions in buffers that contain no EDTA, 5 mM EDTA, 2 mM MgCl_2_, 4 mM ATP, 20 μM PLP, and 1 mM AOAA (PLP-inactivating reagent) at 37°C for 24 h. Data from three technical replicates are averaged and the standard deviations are shown as error bars.


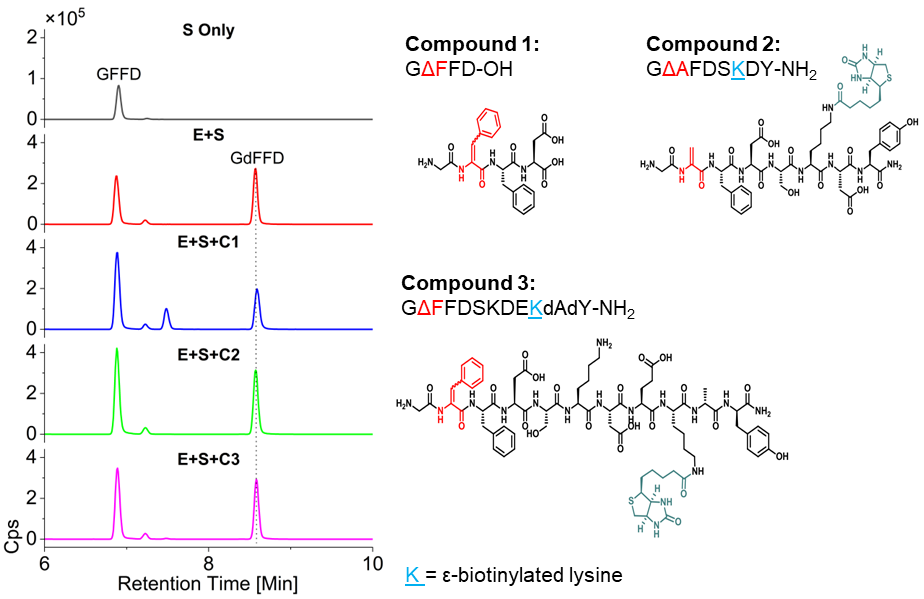


**Figure S3: Effect of synthetic planar substrate analogs on isomerase activity in *A. californica.*** 1st panel: a control of L-substrate incubated with buffer did not show spontaneous isomerization. 2nd panel: a control of L-substrate incubated with an isomerase fraction. D-product was detected post- incubation (arrow). 3rd panel: the planar substrate GΔFFD was incubated with GFFD and isomerase fraction. Isomerase activity appears to be suppressed by the addition of GΔFFD. 4th and 5th panels: addition of the planar substrates GΔAFDSKDYa or GΔFFDSKDEKdAdYa did not produce a strong inhibitory effect on isomerase activity. All substrates were incubated at a concentration of 10 μM, with equivalent amounts of the substrate analog when indicated. “E” refers to Enzyme, “S” for substrate, and C1, C2 and C3 refer to compounds 1, 2 and 3 respectively.

**
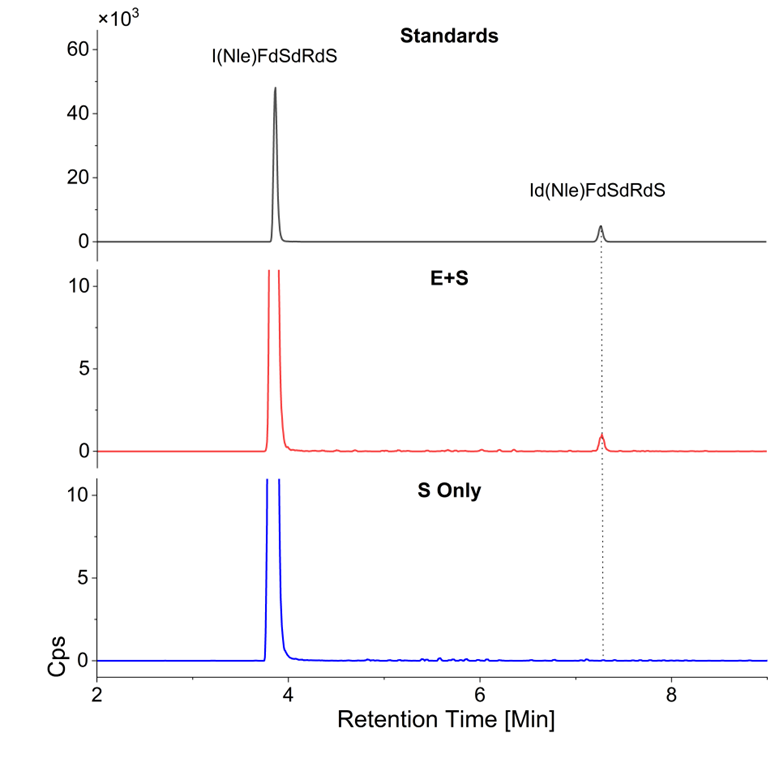
**

**Figure S4: Isomerase activity in mouse heart.** Black trace: MRM chromatogram showing the retention times of synthetic I(Nle)FdSdRdS and I(dNle)FdSdRdS. Red trace: the L- substrate incubated with an enzyme fraction from mouse heart. Blue trace: a control sample of the L-substrate incubated in buffer, showing that isomerization does not occur spontaneously under the assay conditions. “E” refers to Enzyme, “S” for substrate.


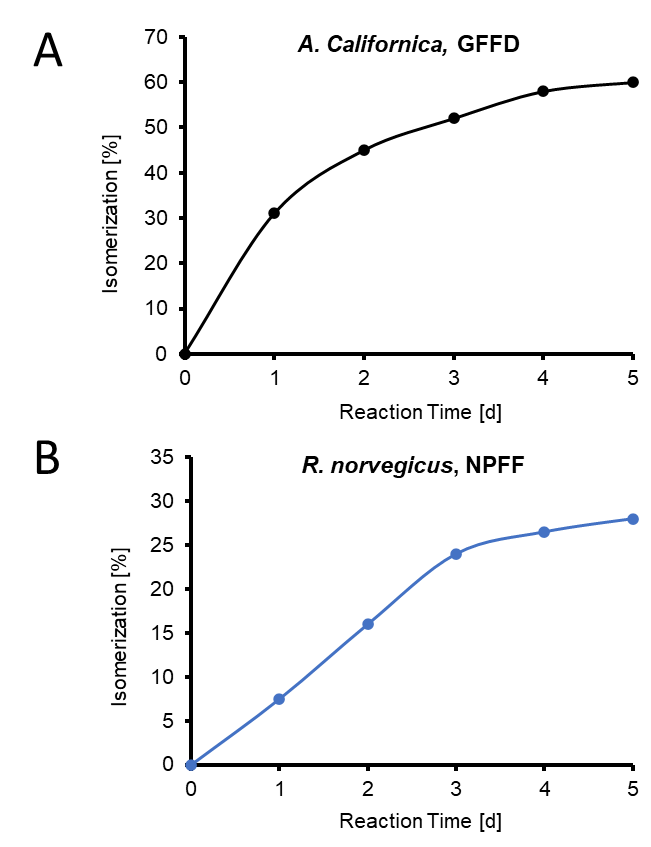


**Figure S5: A reaction time course comparison between *A. californica* and *R. norvegicus* peptide isomerases.** Percent isomerization refers to the total area of isomerized product divided by the total area of substrate in enzyme-free negative controls. **(A)** Conversion of GFFD to GdFFD in fractionated CNS protein homogenate from *A. californica*. GFFD was incubated in an isomerase fraction at 37 °C for 0, 1, 2, 3, 4, and 5 d. **(B)** Conversion of L-NPFF to D-NPFF in fractionated lung protein homogenate from *R. norvegicus*. L-NPFF was incubated in an isomerase fraction at 37 °C for 0, 1, 2, 3, 4, and 5 d.


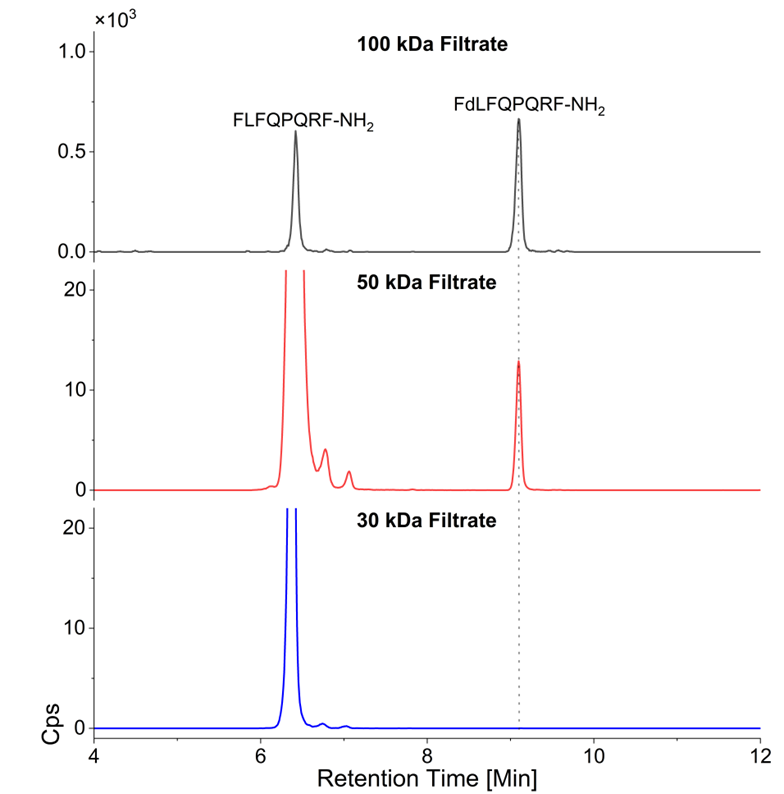


**Figure S6: *R. norvegicus* isomerase activity after centrifugal filtration.** Isomerase activity is present in the filtrate of total protein homogenate from lung tissue. While isomerase-active, evidence of substrate degradation is present due to low signal from both L- and D- peptides using filtrate from a 100-kDa MWCO filter (top black trace). When the 100-kDa MWCO filtrate is applied to a 50-kDa MWCO filter, the filtrate contains isomerase activity without substantial degradation of the L-peptide (middle red trace). Finally, when the 50-kDa MWCO filtrate is applied to a 30-kDa MWCO filter activity is lost in the filtrate (bottom blue trace).
